# Supplementary material for: Random sub-diffusion and capture of genes by the nuclear pore reduces dynamics and coordinates inter-chromosomal movement
Source: eLife. 2021 May 18;10:e66238. doi: 10.7554/eLife.66238 (PMC8195609; doi:10.7554/eLife.66238)
Supplement: Supplementary file 1. [file elife-66238-supp1.docx]

Supplementary file 1. Mean Squared Displacement parameters^†^

| **Locus** | **Medium** | **Strain** | **Γ** | ***R^2^*** | **Figure** |
| --- | --- | --- | --- | --- | --- |
| *URA3*^¥^ | SDC | WT | 0.013 | 0.998 | 2B & D |
| *URA3* | SDC -inositol | WT | 0.014 | 0.997 | 2D |
| *URA3* | SDC -histidine | WT | 0.016 | 0.998 | 2D |
| *HIS4*^¥^ | SDC | WT | 0.021 | 0.998 | 2B & F |
| *HIS4* | SDC -histidine | WT | 0.014 | 0.989 | 2F |
| *INO1*^¥^ | SDC | WT | 0.017 | 0.997 | 2B & E |
| *INO1* | SDC -inositol | WT | 0.015 | 0.963 | 2E |
| *INO1* | SDC | *ino2* | 0.016 | 0.991 | 3E |
| *INO1* | SDC -inositol | *ino2* | 0.017 | 0.994 | 3E |
| *INO1* | SDC | *opi1* | 0.018 | 0.999 | 3F |
| *INO1* | SDC -inositol | *opi1* | 0.018 | 0.983 | 3F |
| *URA3:GRS1* | SDC | WT | 0.009 | 0.987 | 3A |
| *URA3:GRS1* | SDC -inositol | WT | 0.010 | 0.998 | 3A |
| *URA3:GRS1* | SDC | *nup2* | 0.019 | 0.997 | 3C |
| *URA3:GRS1* | SDC -inositol | *nup2* | 0.016 | 0.997 | 3C |
| *URA3:Gcn4BS* | SDC | WT | 0.018 | 0.992 | 3B |
| *URA3:Gcn4BS* | SDC -histidine | WT | 0.013 | 0.999 | 3B |
| *URA3:Gcn4BS* | SDC | *nup2* | 0.018 | 0.999 | 3D |
| *URA3:Gcn4BS* | SDC -histidine | *nup2* | 0.017 | 0.964 | 3D |
| *INO1 (3D acq.)* | SDC | WT | 0.017 | 0.998 | * |
| *INO1 (3D acq.)* | SDC -inositol | WT | 0.015 | 0.967 | * |
| *TSA2*^¥^ | SDC | WT | 0.011 | 0.992 | 2B |
| *GAL2*^¥^ | SDC | WT | 0.012 | 0.993 | 2B |
| *HIS1*^¥^ | SDC | WT | 0.013 | 0.992 | 2B |
| *HIS2*^¥^ | SDC | WT | 0.013 | 0.996 | 2B |
| *HIS5*^¥^ | SDC | WT | 0.011 | 0.992 | 2B |
| *HSP104*^¥^ | SDC | WT | 0.015 | 0.985 | 2B |
| *GAL1*^¥^ | SDC | WT | 0.011 | 0.993 | 2B |
| *TeloVIIIL* | SDC | WT | 0.009 | 0.999 | 2B |
| *TeloXIVL* | SDC | WT | 0.009 | 0.996 | 2B |

^†^ from non-linear fit to MSD(τ) = Γ (τ)^0.52^

^¥^ nucleoplasmic loci from Figure 2B
