# Supplementary material for: Random sub-diffusion and capture of genes by the nuclear pore reduces dynamics and coordinates inter-chromosomal movement
Source: eLife. 2021 May 18;10:e66238. doi: 10.7554/eLife.66238 (PMC8195609; doi:10.7554/eLife.66238)
Supplement: Supplementary file 2. [file elife-66238-supp2.docx]

**Supplementary file 2. Strains used in this study**

| **Name** | **Figures** | **Genotype** |
| --- | --- | --- |
| ASY209 | 2 B,C | *MAT****a*** *ade2-1 can1-100 his3-11,15 TRP1:ER04 LEU2:EGFP-LacI HIS1:p6LacO* |
| ASY210 | 2 B,C | *MAT****a*** *ade2-1 can1-100 his3-11,15 TRP1:ER04 LEU2:EGFP-LacI HIS2:p6LacO* |
| ASY212 | 2B&C | *MAT***a** *ade2-1 can1-100 his3-11,15 leu2-3,112 trp1-1, TRP1: ER04, LEU: EGFP-LacI, HIS5:p6LacO128* |
| BFY_myo3 | 5 supp1 | *MAT****a*** *can1-100 leu2-3,112 ura3-1 HIS3:LacI-GFP TRP1:pER04 INO1:p6LacO128 myo3∆::KanMX* |
| BFY_myo5 | 5 supp1 | *MAT****a*** *can1-100 leu2-3,112 ura3-1 HIS3:LacI-GFP TRP1:pER04 INO1:p6LacO128 myo5∆::KanMX* |
| CEY076 | 1 B,C,H-J; 2 F | *MAT****a*** *ade2-1 can1-100 his3-11,15 ura3-1 TRP1:pER04 LEU2:LacI-GFP HIS4:p6LacO128* |
| CEY135 | 6 B-D; 7 E | *MAT****a****/MATa ade2-1/ade2-1 can1-100/can1-100 his3-11,15/his3-11,15 LEU2:LacI-GFP/leu2-3,112 TRP1:pER04/trp1-1 ura3-1/ura3-1 HIS4:p6LacO128/HIS4:p6LacO128* |
| CEY140 | 6 B-D; 7 G | *MAT****a****/MATa ade2-1/ade2-1 can1-100/can1-100 his3-11,15/his3-11,15 LEU2:LacI-GFP/leu2-3,112 TRP1:pER04/trp1-1 URA3:p6LacO128-GCN4BS:KanMX/ura3-1 HIS4:p6LacO128/HIS4* |
| CEY147 | 3 B | *MAT****a*** *ade2-1 can1-100 his3-11,15 TRP1:pER04 LEU2:LacI-GFP URA3:p6LacO128-GCN4BS* |
| CEY270 | 6 B-D; | *MAT****a****/MATa ade2-1/ade2-1 can1-100/can1-100 his3-11,15/his3-11,15 LEU2:LacI-GFP/leu2-3,112 TRP1:pER04/trp1-1 ura3-1/ura3-1 HIS4:p6LacO128 /HIS4:p6LacO128 gcn4∆::KanMX/gcn4∆::KanMX* |
| CEY271 | 6 B-D; 7 H | *MAT****a****/MATa ade2-1/ade2-1 can1-100/can1-100 his3-11,15/his3-11,15 LEU2:LacI-GFP/leu2-3,112 TRP1:pER04/trp1-1 ura3-1/ura3-1 HIS4:p6LacO128 /HIS4:p6LacO128 gcn4-uORFmt/gcn4∆-uORFmt* |
| CEY391 | 5 E | *MAT****a****/MATa ade2-1/ADE2:pGAL1-GCN4PD-LexA can1-100 his3-11,15/HIS3:LacI-GFP leu2-3,112/LEU2:pGPDmCherry-ER05 trp1-1/trp1-1 URA3:p6LacO128-LexABS:KanMX/URA3:p6LacO128-LexABS:KanMX* |
| DBY008 | 3 F | *MAT****a*** *leu2-3,112 trp1-1 ura3-1 opi1∆::LEU2 SEC63-13myc:KanMX INO1:p6LacO128 His3:LacI-GFP* |
| DBY1840 | 3 D | *MAT****a*** *ade2-1 can1-100 his3-11,15 TRP1:pER04 LEU2:LacI-GFP URA3:p6LacO128-GCN4BS nup2-cr* |
| DBY471 | 2 A,B | *MAT****a*** *ade2-1 can1-100 his3-11,15 leu2-3,112 trp1-1 ura3-1 LEU2:LacI-GFP SPC29-RFP:Hyg* |
| DBY475 | 2B&C | *MAT***a** *ade2-1 can1-100 his3-11,15 leu2-3,112 trp1-1 ura3-1 LEU2:LacI-GFP SPC29-RFP:Hyg HSP104:pFS2913* |
| DBY477 | 2B&C | *MAT***a** *ade2-1 can1-100 his3-11,15 leu2-3,112 trp1-1 LEU2:LacI-GFP SPC29-RFP:Hyg URA3:p6LacO128* |
| DBY507 | 2 B,C | *MATa ade2-1 can1-100 his3-11,15 trp1-1 ura3-1 LEU2:LacI-GFP SPC29-RFP:Hyg TSA2:p6LacO128* |
| **Name** | **Figures** | **Genotype** |
| DBY508 | 2 B,C | *MATa ade2-1 can1-100 his3-11,15 trp1-1 ura3-1 LEU2:LacI-GFP SPC29-RFP:Hyg GAL2:p6LacO128* |
| DBY509 | 2 B,C | *MATa ade2-1 can1-100 his3-11,15 trp1-1 ura3-1 LEU2:LacI-GFP SPC29-RFP:Hyg TeloVIIIL:LacO-TRP1* |
| DBY510 | 2 B,C | *MATa ade2-1 can1-100 his3-11,15 trp1-1 ura3-1 LEU2:LacI-GFP SPC29-RFP:Hyg TeloXIVL:LacO-TRP1* |
| DBY598 | 6 B-D; 7 K | *MAT****a****/MATa ade2-1/ade2-1 can1-100/can1-100 HIS3:LacI-GFP/his3-11,15 LEU2:LacI-GFP/leu2-3,112 TRP1:pER04/TRP1:pER04 ura3-1/ura3-1 LEU2:LacI-GFP TRP1:pER04 INO1:p6LacO128/INO1:p6LacO128* |
| JBY499 | 3 C | *MAT****a*** *ade2-1 can1-100 leu2-3,112 trp1-1 URA3:p6LacO128 HIS3:LacI-GFP nup2∆::KanMX* |
| MSY023 | 2 A,B | *MAT****a*** *ade2-1 can1-100 his3-11,15 leu2-3,112 trp1-1 ura3-1 LEU2:pAG415GPD-EGFP-µNS* |
| MSY027 | 5 E-G,I | *ade2-1/ade2-1 can1-100/can1-100 his3-11,15/HIS3:LacI-GFP,15 LEU2:CIB1-Gcn4PD/LEU2:Pho88-mCherry TRP1:CRY2-LexADBD/trp1-1 ura3-1/URA3:p6LacO KanMX:LexABS* |
| MSY028 | 5 E-G,I | *MAT****a****/MATa ade2-1/ade2-1 can1-100/can1-100 his3-11,15/HIS3:LacI-GFP,15 LEU2:CIB1-Gcn4mutPD/LEU2:Pho88-mCherry TRP1:CRY2-LexADBD/trp1-1 ura3-1/URA3:p6LacO KanMX:LexABS* |
| MSY042 | 6 B-D | *MAT****a****/MATa ade2-1/ade2-1 can1-100/can1-100 his3-11,15/his3-11,15 LEU2:LacI-GFP/leu2-3,112 TRP1:pER04/trp1-1 URA3:p6LacO128/URA3:p6LacO128* |
| MSY043 | 6 B-D; 7 J | *MAT****a****/MATa ade2-1/ade2-1 can1-100/can1-100 his3-11,15/his3-11,15 LEU2:LacI-GFP/leu2-3,112 TRP1:pER04/trp1-1 URA3:p6LacO128/ura3-1 HIS4:p6LacO128/HIS4* |
| NDY003 | 1 D,F,H-J; 2 E | *MAT****a*** *can1-100 leu2-3,112 ura3-1 HIS3:LacI-GFP TRP1:pER04 INO1:p6LacO128* |
| NDY005 | 1 D | *MAT****a*** *can1-100 leu2-3,112 ura3-1 HIS3:LacI-GFP TRP1:pER04 INO1:p6LacO128* |
| NDY007 | 1 D,G-J; 3A; 5 C,F,G,I | *MAT****a*** *can1-100 leu2-3,112 ura3-1 HIS3:LacI-GFP TRP1:pER04 URA3:p6LacO120-GRSI* |
| NDY002 | 1 B,D,E,H-J; 2 D; 5 C,F,G,I | *MAT****a*** *can1-100 leu2-3,112 ura3-1 HIS3:LacI-GFP TRP1:pER04 LacO:URA3* |
| SAY53 | 3 E | *MAT****a*** *ade2-1 can1-100 his3-11,15 leu2-3,112 trp1-1 ura3-1 INO1:p6LacO128 HIS3:LacI-GFP ino2∆:: kanMX6* |
